# Extracapsular Dissection for Small Benign Tumors of Parotid Gland: A Case Report on Contemporary Technique

**DOI:** 10.7759/cureus.17007

**Published:** 2021-08-08

**Authors:** Ravi Raja K Saripalli, Leela Subhashini C Alluri, Ananthnag Jakkula, Sai Sarat Yadavilli

**Affiliations:** 1 Department of Oral and Maxillofacial Surgery, St. Joseph Dental College, Eluru, IND; 2 Periodontics, Private Practice, Oklahoma, USA; 3 Department of Oral and Maxillofacial Surgery, GSL Dental College & Hospital, Rajahmundry, IND

**Keywords:** benign parotid tumors, extracapsular dissection, superficial parotidectomy, salivary gland tumor, pleomorphic adenomas

## Abstract

Salivary gland tumor looms as painless enlarging mass which may embrace in both major or minor glands. Pleomorphic adenoma (PA) accord about 40-70% of all salivary gland tumors, where Warthin tumor, basal cell adenoma (BCA), adenoid cystic carcinoma (ACC), and sebaceous tumors have a strong predilection for major salivary gland. However, polymorphous low-grade adenocarcinoma (PLGA) has a marked predilection for the minor salivary gland. We present a case of PA in a 26-year-old male patient that has been successfully managed by extracapsular dissection (ECD) without any post-operative complications.

## Introduction

Pleomorphic adenoma (PA) is a true neoplasm that will continue to grow or regrow, if not completely removed [1,2]. It is one of the most commonly seen salivary gland neoplasms accounting for 53-77% of parotid tumors, and 1.5-23% of cases of recurrent PA undergo malignant changes [3,4]. Around 80% of all PAs develop from the parotid gland superficial lobe, presenting as firm and freely movable mass [3]. It usually goes unrecognized for many years, when it arises from the parotid gland's deep lobe until the size creates dysphagia or gagging symptoms [3,5]. The majority of these tumors are slow-growing, painless, and usually lateral to the facial nerve. However, a few long-standing cases may show the malignant transformation. Pain, rapid growth, and facial paresis are usually indicative of malignant transformations. Various surgical options have been described for benign parotid tumors in the literature based on the extent of the tumor. Extracapsular dissection (ECD) is one of the least invasive techniques for the successful management of superficial parotid tumors.

## Case presentation

**Figure 1:**
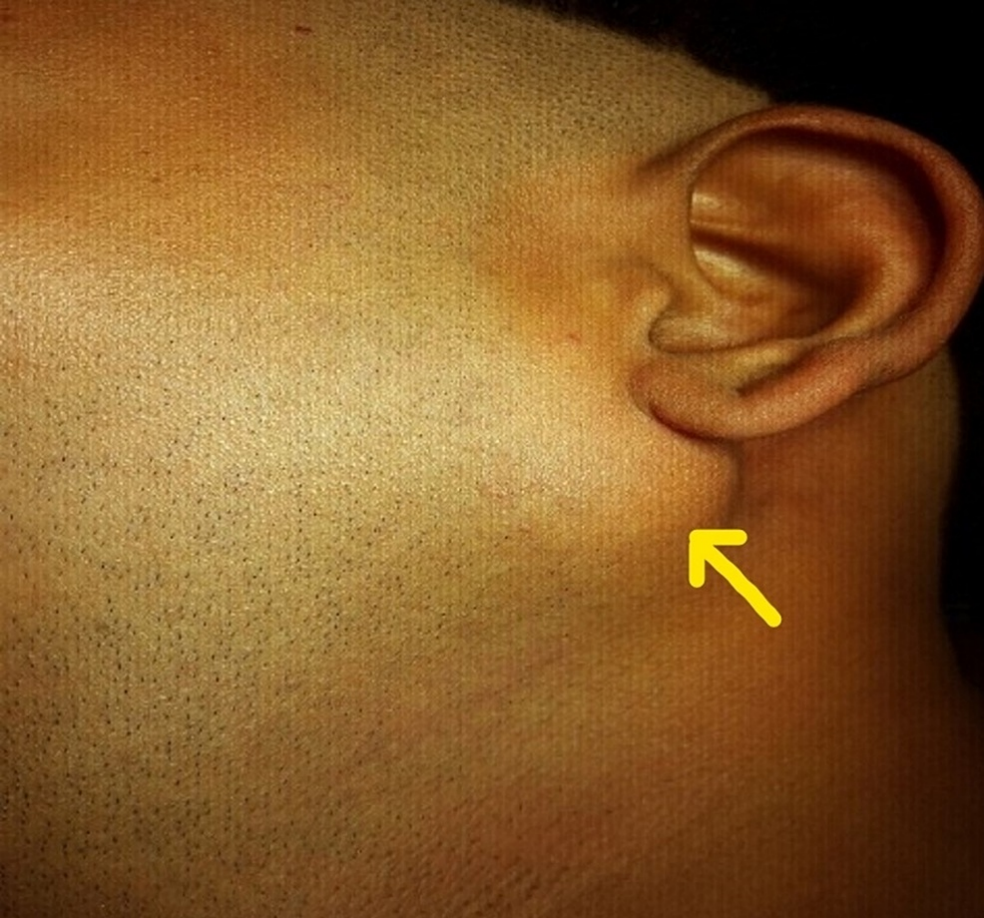
Preoperative extraoral view.

A 26-year-old healthy male with no significant medical history reported a painless swelling behind the left ear, progressively increasing in six months. On clinical examination, a firm, freely movable mass roughly measuring 3 x 2 cm in size was found just below the left pinna of the ear (Figure 1).

It had no features of facial paresis. The swelling was provisionally considered to be originated from the left parotid gland. The patient priorly consulted an ENT surgeon at a private clinic and got fine needle aspiration cytology (FNAC) and MRI performed.

Investigations

On examination of the already performed investigation, the following observations were made. FNAC smears revealed rich cell yield comprising sheets and clusters of salivary gland epithelial cells with amphophilic mucomyxoid material in the background. In addition, the cytological findings are suggestive of PA of the left parotid gland.

**Figure 2:**
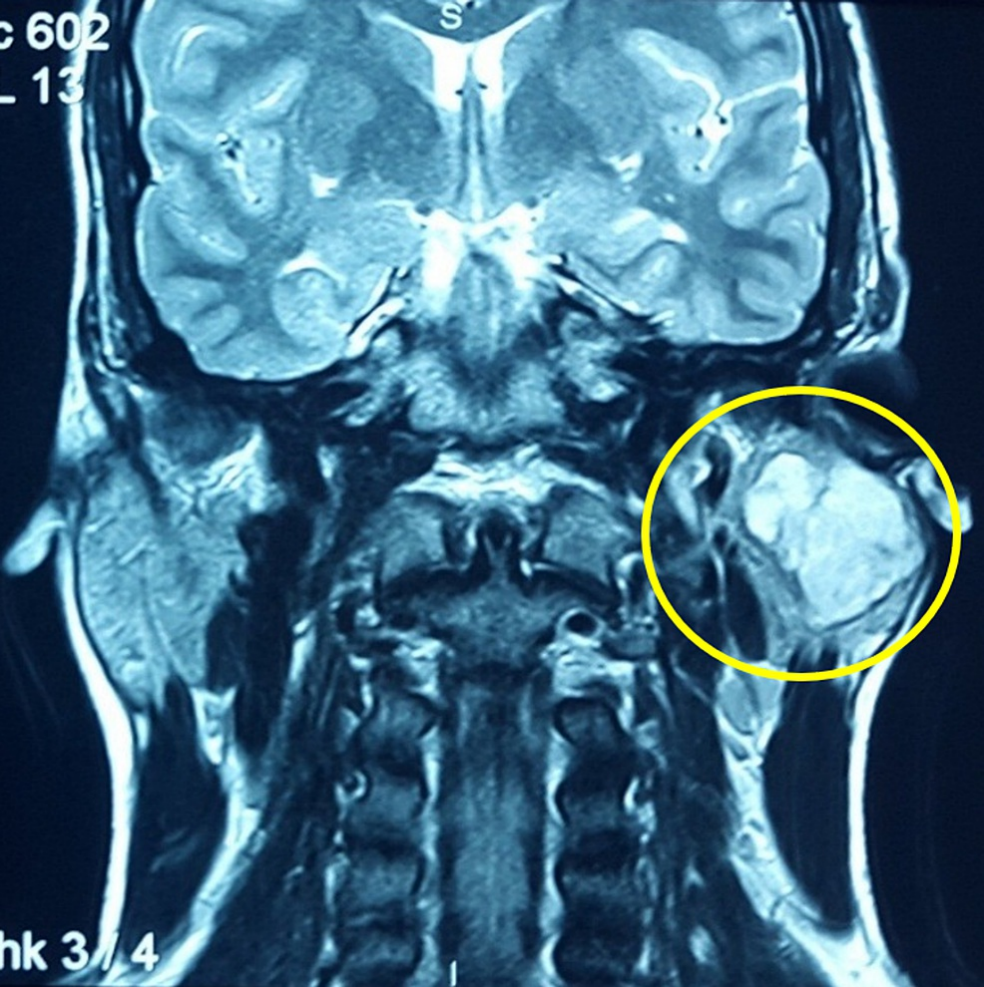
Preoperative coronal sections of the MRI scan.

**Figure 3:**
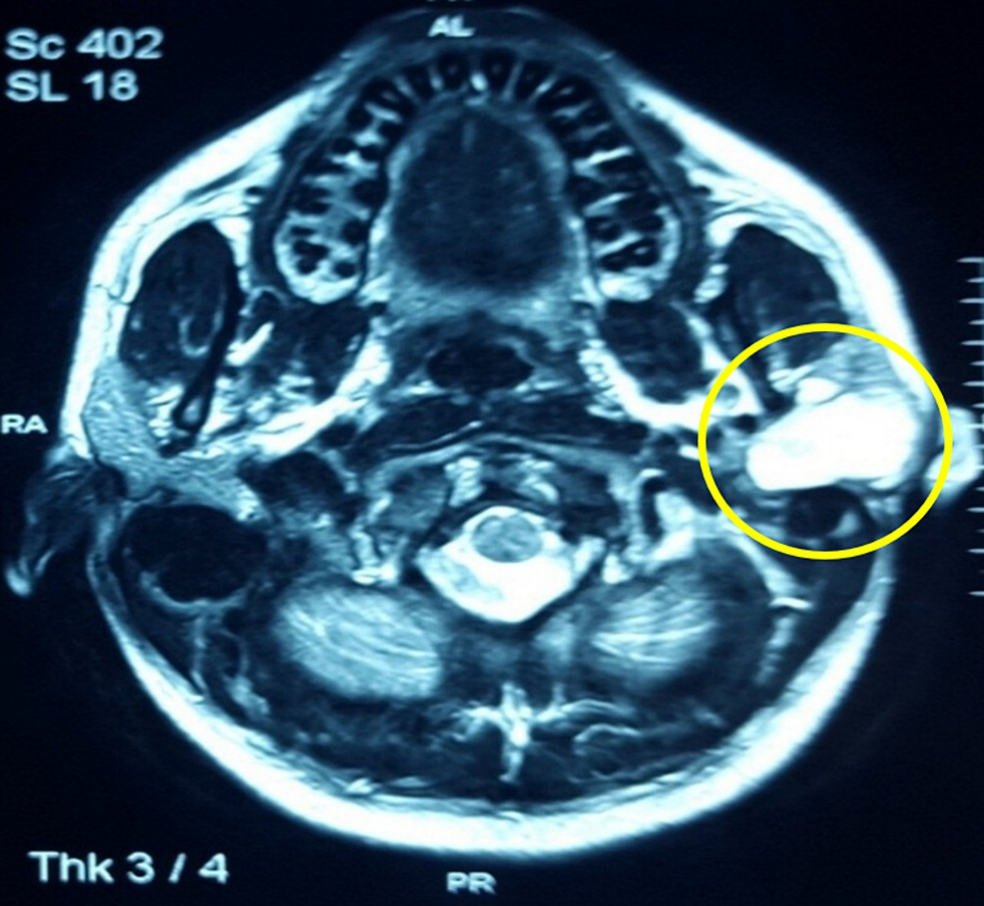
Preoperative axial sections of the MRI scan.

MRI scan was performed for the left parotid gland, which showed a lobulated, well-defined T2 and short inversion time inversion-recovery (STIR) hyperintense mass involving the superficial lobe of the left parotid gland with hypointense separations noted within the gland. The coronal (Figure 2) and axial (Figure 3) sections of the MRI showed the tumor's extent. Vascular structures are normal, and there is no cervical lymphadenopathy. These features are suggestive of a benign tumor, probably a PA of the parotid gland.

Surgical Technique

**Figure 4:**
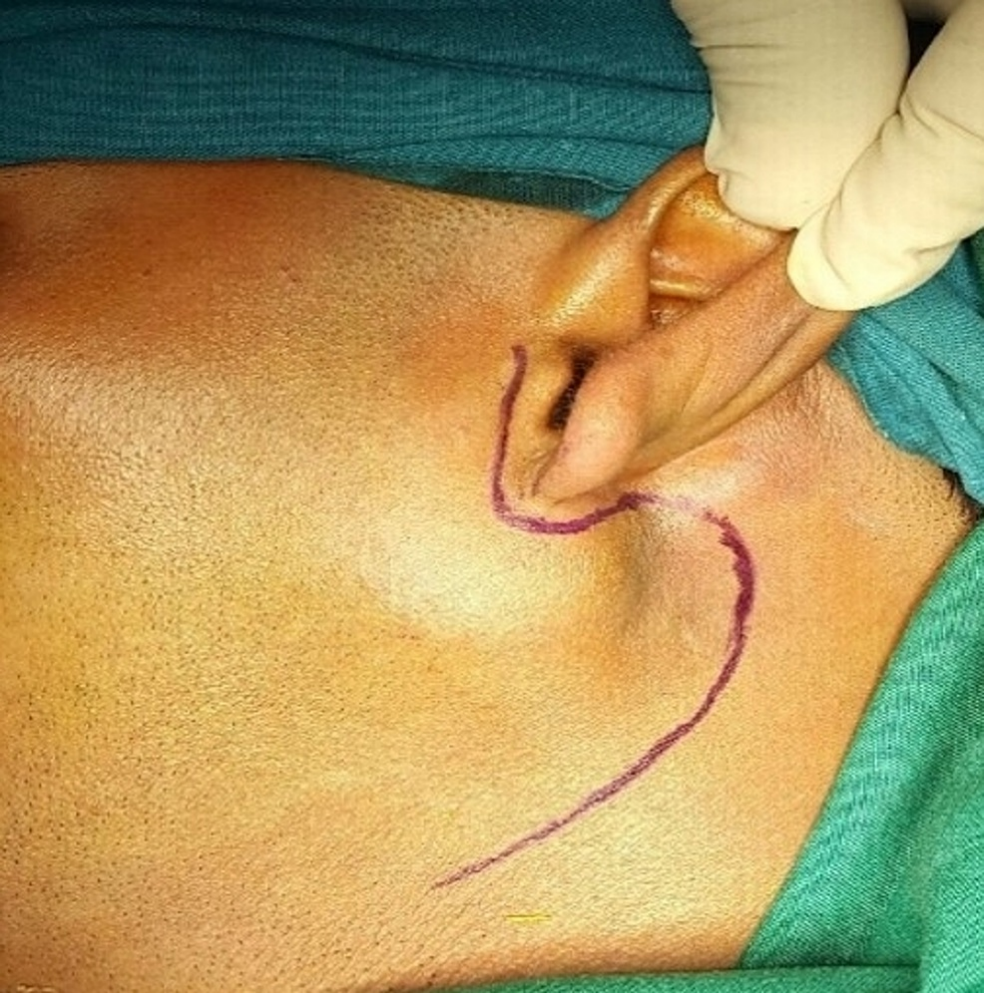
View showing a modified Blair's (lazy S-shaped) incision. Marking was given around the ear.

After obtaining informed consent verbally, written, and signed by the patient, ECD and excision of the lesion were planned contrary to the traditional superficial parotidectomy. However, the possible necessity of superficial parotidectomy was also discussed and explained to the patient. Besides, the possible postoperative sequelae were explained to the patient. A modified Blair's (lazy S-shaped) incision was given around the ear (Figure 4).

**Figure 5:**
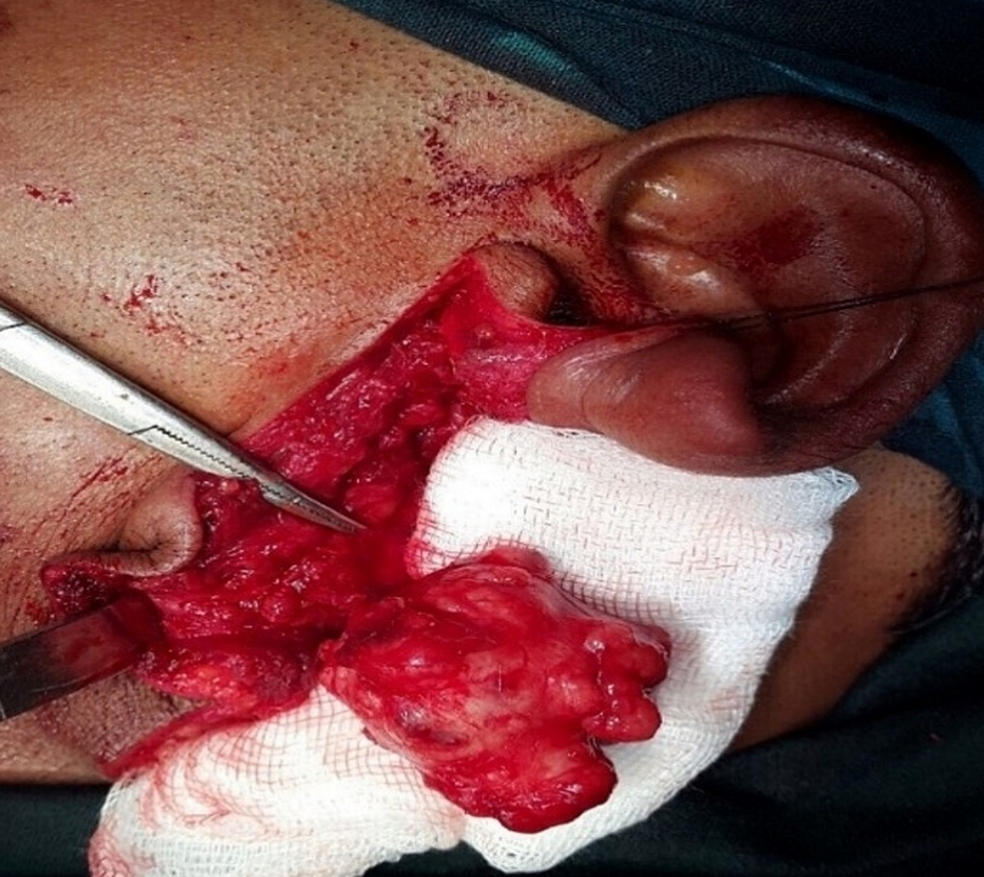
Intraoperative photograph showing the removal of the tumor with extracapsular dissection.

A small incision was made in the superficial muscular aponeurotic system (SMAS) and displayed in one layer to permit primary closure following removing the tumor. The nerve monitor was utilized during the procedure to decrease the odds of injury to the facial nerve, although none of the facial nerve branches were encountered. The tumor mass was excised in toto (Figure 5).

**Figure 6:**
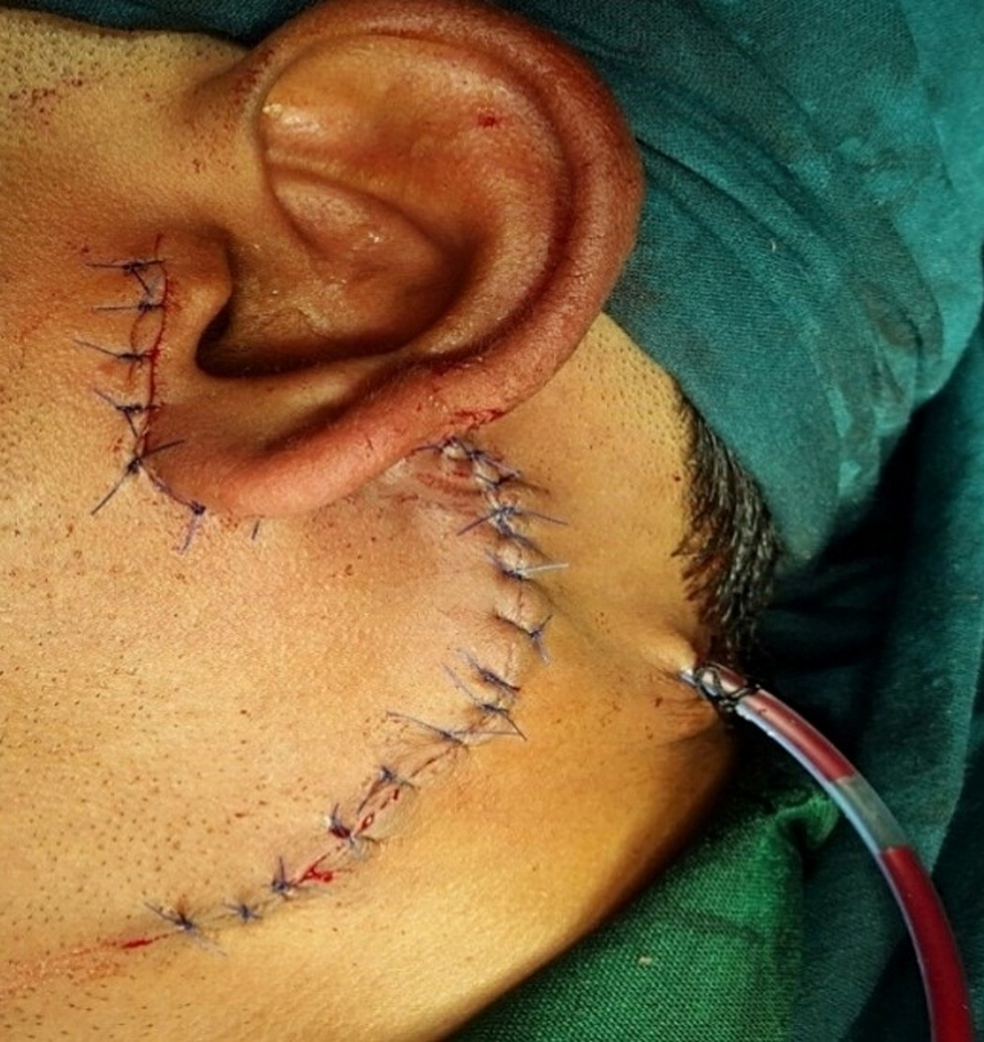
View of the patient immediately after surgery.

Wound closure was done using 3.0 vicryl and 4.0 prolene. The vacuum drain was placed in the post-surgical defect and removed after 48 hours (Figure 6).

**Figure 7:**
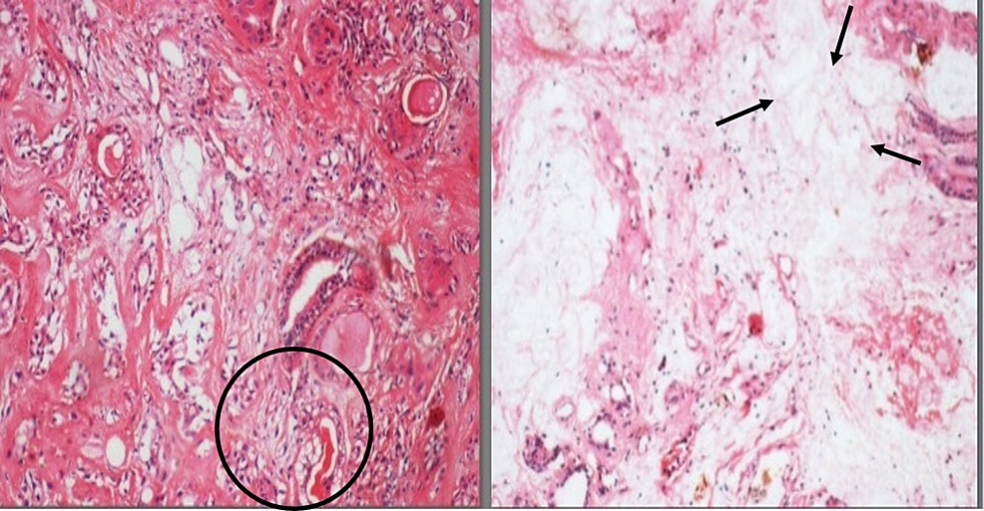
Histopathology slide showing the features of pleomorphic adenoma. Figure 7A: The left side showing a duct-like pattern with eosinophilic coagulum. Figure 7B: The right side showing the myxoid area in the histopathologic area.

The post-operative period was uneventful, with no signs of facial paresis. The wound healing at the operated site was satisfactory. There were no symptoms of sialocele, no evidence of loss of sensory or motor function, and no gustatory sweating symptoms following surgery. The follow-up was done weekly for two months and then followed for three years at a six-month interval period. The excised tumor mass was sent for a histopathological examination. Histopathological examination showed a well-capsulated, highly cellular mass composing of both epithelial and stromal components. Epithelial tumor cells were arranged in duct-like patterns with eosinophilic coagulum in the duct center, and focal areas showed keratin pearls formation. The myxoid areas were also seen. Based on the above features, a confirmed diagnosis of "PA" was given (Figures 7A-7B).

## Discussion

WHO (1972) stated that PA is characterized by a mixed appearance containing the epithelial components combined with the mucoid, chondroid, and myxoid components. Though it shows numerous histological features, it is usually considered to be benign [1,6]. Regardless of its site's origin, the PA emerges as a slow-growing, painless, and firm mass. At any age, the tumor may occur and is often common in young adults between 30 and 50 with a slight female predilection. The benign tumor is not responsible for the facial nerve paresis. However, facial weakness not contributed from the previous surgery should be considered malignant until proven otherwise [7]. A CT scan, ultrasound, or MRI scan is essential to confirm the parotid gland's tumor location. The tumor is usually well-circumscribed and encapsulated [8,9]. However, the capsule can be incomplete and show infiltration by the tumor cells. This absence of complete encapsulation is often frequent for minor salivary gland tumors. Investigations with FNAC and histopathology, our case exhibited mixed epithelial and mesenchymal pattern components. The myoepithelial cell is thought to be the cell of origin capable of producing such a variety of morphological diversity. Histopathologically, PA consists of the duct-like structure made up of epithelial cells with the association of non-ductal cells, i.e., myoepithelial cells, within a heterogeneous stromal background myxoid, chondroid, and osseous differentiated structure [3,10]. The parotid gland neoplasms' differential diagnosis needs to include benign lesions like Warthin tumor, myoepithelioma, and basal cell adenoma (BCA). Additionally, malignant salivary gland tumors, including mucoepidermoid, adenoid cystic, and acinic cell carcinomas, need to be considered. Non-salivary gland neoplasms known to arise in the parotid gland include hemangioma, lymphangioma, lymphoma, and lipomas within the parotid lymph nodes [3].

As PA evince varied histopathological features, it may be confused with other salivary gland tumors like myoepithelioma, adenoid cystic carcinoma (ACC), mucoepidermoid carcinoma (MEC), and BCA. Due to histopathological and cytogenetic similarities, myoepitheliomas can be misdiagnosed with PA; but the former lacks typical glandular differentiation and chondromyxoid areas. ACC shows both epithelial and myoepithelial cell differentiation; however, infiltrative growth patterns with the mitotic figure and perineural invasion are the salient features of ACC. Intermediate cells in MEC show histopathological similarity to non-ductal cells, i.e., myoepithelial cells of PA; however, former cells lack myxochondroid stroma formation. BCA, earlier thought of as a subtype of PA, histologically shows similar features; however, BCA shows the absence of myxochondroid stroma component [1,11]. In our present case, the histopathological features revealed significant characteristics of PA with well-capsulated islands of epithelial and rounded myoepithelial cells, a cellular mass of sheets, and ductal architecture along with myxomatous background was apparent. 

The traditional method for surgical management of parotid tumors varies from superficial parotidectomy to total parotidectomy, depending on the extent of the lesion. Complete superficial parotidectomy is the excision of the whole parotid gland adjacent to the facial nerve. In contrast, partial superficial parotidectomy is only limited to dissection depending on the involvement of the tumor, along with 1-2 cm of normal parotid gland tissue adjacent to the neoplasm after identification of facial nerve trunk. A total parotidectomy involves removing all the parotid gland tissue, both medial and lateral, to the facial nerve. In contrast, ECD carries out a precise blunt prosection across the parotid gland tissue by performing a cruciate incision directly above the tumor [12]. ECD is a good choice for small mobile superficial tumors and is gaining more popularity due to low morbidity [13]. Understanding the various philosophies beyond superficial parotidectomy and ECD is essential. Parotidectomy usually follows the peripheral facial plexus plane, which means facial nerve dissection surgery [14]. ECD prevents a formal nerve dissection in preference to a careful dissection around the tumor itself [12]. Thus, facial nerve examination is definite during ECD. As the tumor is small in size and superficial in our case, ECD was carried out without any intraoperative or postoperative sequelae. However, ECD might have positive resection margins, and recurrent disease was significantly more frequent after ECD than superficial parotidectomy [12]. So careful selection of the patient and the surgeon's experience will determine the outcome of ECD for superficial parotidectomy.

## Conclusions

The principles following superficial parotidectomy and ECD are distinct. The ECD is reserved for cases where the tumor is benign, small in size, superficial, and freely movable. ECD is gaining popularity because of low morbidity; besides, facial nerve monitoring should be done during the procedure to prevent any complications.
